# Ginsenoside-Rb1 targets chemotherapy-resistant ovarian cancer stem cells via simultaneous inhibition of Wnt/β-catenin signaling and epithelial-to-mesenchymal transition

**DOI:** 10.18632/oncotarget.13071

**Published:** 2016-11-04

**Authors:** Shan Deng, Chris Kong Chu Wong, Hung-Cheng Lai, Alice Sze Tsai Wong

**Affiliations:** ^1^ School of Biological Sciences, University of Hong Kong, Hong Kong; ^2^ Department of Biology, Hong Kong Baptist University, Kowloon Tong, Hong Kong; ^3^ Department of Obstetrics and Gynecology, Shuang Ho Hospital, Taipei Medical University, Taipei, Taiwan

**Keywords:** Wnt/β-catenin signaling, ovarian cancer, chemoresistance, cancer stem cells, ginsenoside

## Abstract

Chemoresistance is a major clinical problem compromising the successful treatment of cancer. One exciting approach is the eradication of cancer stem/tumor-initiating cells (jointly CSCs), which account for tumor initiation, progression, and drug resistance. Here we show for the first time, with mechanism-based evidence, that ginsenoside-Rb1, a natural saponin isolated from the rhizome of *Panax quinquefolius* and *notoginseng*, exhibits potent cytotoxicity on CSCs. Rb1 and its metabolite compound K could effectively suppress CSC self-renewal without regrowth. Rb1 and compound K treatment also sensitized the CSCs to clinically relevant doses of cisplatin and paclitaxel. These effects were associated with the Wnt/β-catenin signaling pathway by downregulating β-catenin/T-cell factor-dependent transcription and expression of its target genes ATP-binding cassette G2 and P-glycoprotein. We also identified reversal of epithelial-to-mesenchymal transition as a new player in the Rb1 and compound K-mediated inhibition of CSCs. Rb1 and compound K treatment also inhibited the self-renewal of CSCs derived from ovarian carcinoma patients as well as in xenograft tumor model. Moreover, we did not observe toxicity in response to doses of Rb1 and compound K that produced an anti-CSC effect. Therefore, Rb1 should be explored further as a promising nutraceutical prototype of treating refractory tumors.

## INTRODUCTION

Chemotherapy has been the mainstay of cancer treatment for the past several decades. Yet, there is little evidence for progress in reducing cancer mortality, because most of the patients ultimately relapse. The recent discovery of cancer stem/tumor-initiating cells (jointly CSCs) has changed our view of carcinogenesis and chemoresistance [1]. In contrast to other tumor cells, CSCs represent a subpopulation that possesses the ability to self-renew and differentiate, properties that render these cells particularly resistant to therapy. As such, it is conceivable that the relapse after remission is likely due to the inability of current chemotherapeutic drugs to kill CSCs. Despite the tumor bulk elimination, CSCs are capable of reproducing the entire tumor. Thus, strategies aiming at targeting CSCs represent rational approaches for cancer prevention and treatment. Plant-derived small molecules that exhibit potent antitumor activity but low toxicity and limited side effects could be promising new treatment modalities.

Therapeutic values of ginseng (the rhizome of *Panax* ginseng), a key ingredient in Chinese traditional medicine, have been recognized for >1,000 years [2]. It is now one of the most popular alternative medicines widely consumed worldwide and appears in the pharmacopeias of several countries, including the United States and Europe and employed for cancer, diabetes, and cardiovascular concerns. In recent years, ginseng has gained considered attention for the prevention and/or treatment of cancers in epidemiological, basic, and clinical research. This is supported by the findings on its ability to reduce the incidence of carcinogen-induced tumors in mice and to inhibit the growth of tumor cells *in vitro* [3]. A dose-dependent decrease in the risk of cancer as a regular consumption of ginseng in both prospective and case-control studies and a better survival rate and a greater quality of life are also noted [4]. Notably, such effects are not observed in patients using other Chinese traditional medicine [5]. This opens up the exciting possibility of ginseng as an important drug, emphasizing the importance to uncover the exact component(s) in the ginseng extract that contribute to the antitumor function and evaluate its pharmacological action.

Emerging evidence shows that nonpeptide small molecules, such as saponins, exhibit potent cytotoxicity, with great potential to be developed as chemotherapeutic agents [6]. A notable saponin isolated from *P. quinquefolius* and *notoginseng* is ginsenoside-Rb1, which constitutes 0.37-0.5% of ginseng extract [7, 8]. Most (∼70%) orally administered Rb1 is metabolized by intestinal bacteria to its final derivative 20-O-β-D-glucopyranosyl-20(S)-protopanaxadiol (also called compound K) [9]. Compound K is reported to be easily absorbed and sustained longer in the human body. Nevertheless, the key targets of ginsenoside Rb1 and its metabolite compound K have not been explored. Nor is it clear about the molecular mechanisms.

In this study, we show for the first time that Rb1 and its metabolite compound K specifically target the formation and expansion of CSCs. We further provide evidence that Rb1 and compound K can chemosensitize CSCs to clinical anticancer drugs cisplatin and paclitaxel, inducing a synergistic cytotoxicity via Wnt/β-catenin signaling and epithelial-to-mesenchymal transition (EMT) regulation, both attractive targets for cancer treatment.

## RESULTS

### Cytotoxic and anti-proliferative effects of Rb1 and its metabolite compound K on ovarian CSCs

**Figure 1:**
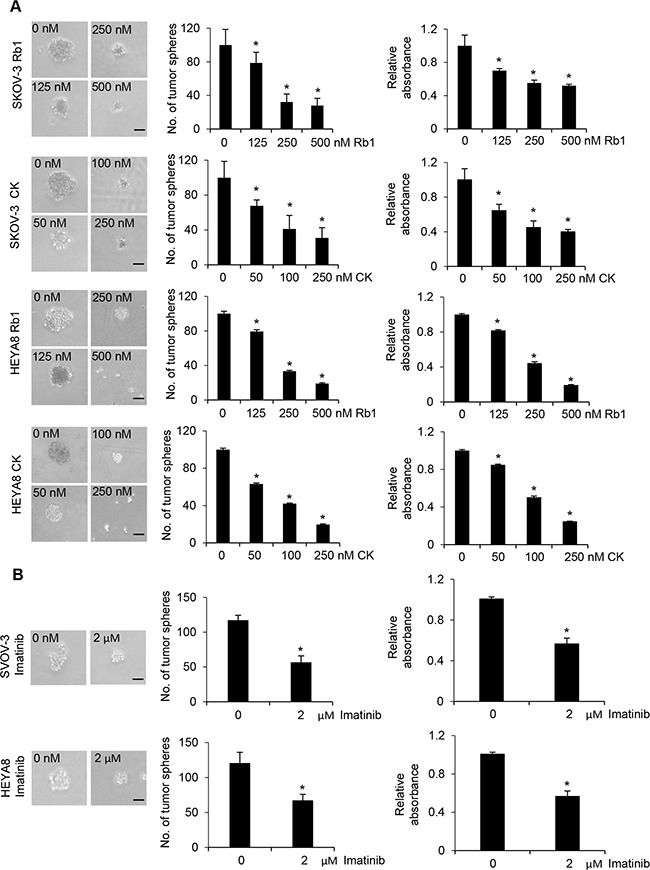
Rb1 and its metabolite compound K inhibit self-renewal and growth of CSCs **A**. The number of tumor spheres generated were photographed (left) and counted (right). In parallel experiments, cell viability was determined by MTT assay. The absorbance of wells not exposed to Rb1 or compound K (CK) treatment was arbitrarily set as 1, and cell growth after Rb1 or CK treatment was expressed as the fold changes compared to the control. **B**. Cell viability was determined after imatinib treatment. Bar, 50 μm. Experiments were repeated three times, and data are shown as mean ± SD. *, *P* < 0.05 vs control.

**Figure 2:**
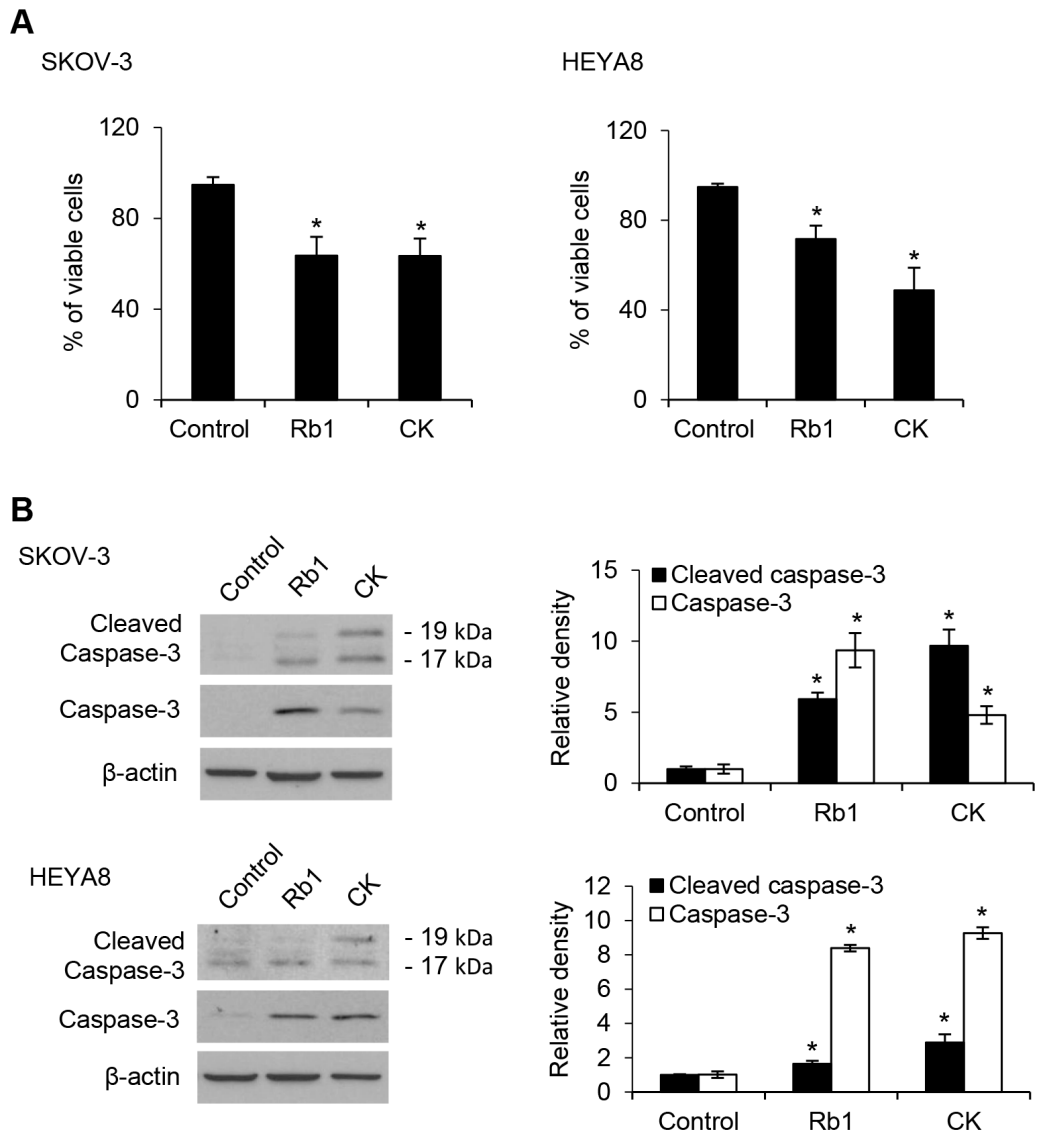
Rb1 and its metabolite compound K induce apoptosis of CSCs **A**. Viable spheres were counted using trypan blue after Rb1 or CK treatment. **B**. The expression of caspase-3 and the active (cleaved) proapoptotic gene caspase 3 were determined by Western blot after Rb1 or compound K treatment. Experiments were repeated three times, and data are shown as mean ± SD. *, *P* < 0.05 vs control.

**Figure 3:**
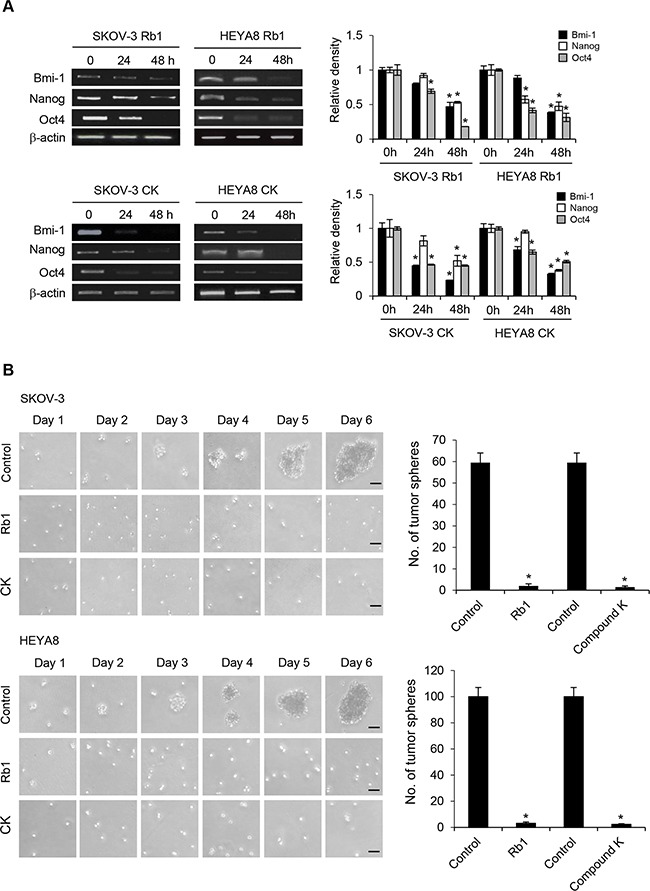
No regrowth/relapse in Rb1- or compound K-treated SKOV-3 and HEYA8 spheroids **A**. Expression of CSC markers Bmi-1, Nanog, and Oct4 after Rb1 or CK treatment. β-actin serves as an internal control. The signal intensity was determined by densitometry. **B**. SKOV-3 and HEYA8 spheroids untreated or treated with Rb1 or compound K (CK) were plated as secondary spheroids. Representative views of secondary spheroids imaged until day 6 (right). The number of tumor spheres generated was photographed and counted (left). Bar, 50 μm. Experiments were repeated three times, and data are shown as mean ± SD. *, *P* < 0.05 vs control.

Ovarian cancer is a highly chemoresistant cancer that is rapidly fatal and most patients will develop tumor recurrence and succumb to chemoresistant disease. Thus, it provides an excellent model to identify the mechanisms required for this drug resistance. Using a functional enrichment strategy based on the self-renewal ability of CSCs to grow as nonadherent spheres under stem-cell-selective condition which recapitulates advanced stages of ovarian carcinoma cells in malignant ascites, we have successfully identified CSCs in ovarian cancer cell lines and cancerous ovarian tissues [10]. Here we first investigated the possible cytotoxic effect of Rb1 and its metabolite compound K on SKOV-3 and HEYA8 CSCs. Figure 1A shows dose-dependent effect of Rb1 and compound K on both line-derived CSCs on tumor sphere formation and growth. In addition, the spheres formed upon Rb1 or compound K treatment were smaller compared with the control (Figure 1). Both Rb1 and compound K suppressed tumor cell survival (Figure 1A). The LC_50_ (the concentration that leads to 50% survival) were 250 nM for Rb1 and 100 nM compound K in SKOV-3 and 230 nM for Rb1 and 125 nM for compound K in HEYA8, respectively. A clinically relevant dose of imatinib also gave similar results (Figure 1B) [10]. In accordance with this, trypan blue exclusion assay showed a 1.5-fold increase in cell death for Rb1 and compound K in SKOV-3 and a 1.4- and 2-fold increase in HEYA8, respectively (Figure 2A). Furthermore, Rb1 and compound K displayed a significant 5.9- and 9.6-fold in SKOV-3 and 1.6 and 2.9- fold in HEYA8 cells increase in apoptosis as revealed by the expression of the active (cleaved) caspase 3 (Figure 2B), suggesting that apoptosis may account for this loss of cell viability. Subsequently, CSCs were treated with 250 nM Rb1 and 125 nM compound K for different periods of time (0, 24, and 48 h). We evaluated CSC marker expression in response to Rb1 or compound K treatment. Three different markers of ovarian CSCs, Bmi-1, Nanog, and Oct4, were tested. Rb1 or compound K depleted all these markers expression in a time-dependent manner, with the maximal effects observed 48 hours following treatment (Figure 3A), reflecting Rb1- and compound K-dependent inhibition of CSC self-renewal and growth of chemotherapy-resistant CSCs.

### No regrowth (relapse) upon Rb1 and its metabolite compound K treatment

Because sphere forming capabilities in serial passages is an indirect marker in support of stem cell renewal, we further determined the effect of Rb1 and compound K in relapse experiment. SKOV-3 and HEYA8 primary spheroids treated with either control vehicle, 250 nM Rb1, or 125 nM compound K were dissociated and replated as secondary spheroids. By day 2, cells from the control group started growing as secondary spheroids, whereas Rb1- or compound K-treated cells did not (Figure 3B). For approximately 6 days, secondary spheroids did not form from Rb1- or compound K-treated samples, whereas control samples developed dense secondary/tertiary spheroids (Figure 3B). Furthermore, the removal of these compounds did not restore the ability of both SKOV-3 and HEYA8 to form spheroids upon serial passage (data not shown). These results suggest that Rb1 and compound K inhibit CSC self-renewal, and that this process is irreversible.

### Rb1 and its metabolite compound K reduce CSC resistance to chemotherapeutic drugs

**Figure 4:**
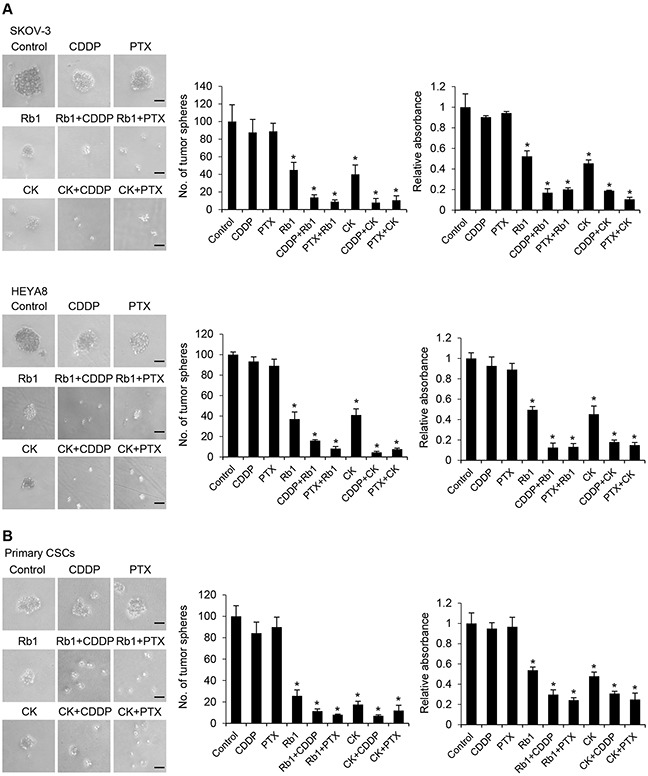
Rb1 and its metabolite compound K reduce chemoresistance of CSCs **A**. SKOV-3 or HEYA8 CSCs or **B**. CSCs isolated from primary ovarian tumors were untreated or treated with cisplatin (CDDP; 50 μM) or paclitaxel (PTX; 100 nM) alone or in combination with Rb1 (250 nM) or compound K (CK) (125 nM) for 24 h. The number of tumor spheres generated were photographed (left) and counted (right). Bar, 50 μm. In parallel experiments, cell viability was determined by MTT assay. The absorbance of wells not exposed to Rb1 or CK treatment was arbitrarily set as 1, and cell growth after Rb1 or CK treatment was expressed as the fold changes compared to the control. Experiments were repeated three times, and data are shown as mean ± SD. *, *P* < 0.05 vs control.

The higher drug resistance of CSCs encouraged us to assess whether Rb1 and compound K will be effective in increasing the treatment efficacy. Rb1 or compound K was used alone or in combination with cisplatin/paclitaxel, the two front-line chemotherapeutic agents currently used for treating unresectable ovarian cancer. We found cisplatin and paclitaxel at clinically relevant does of 50 μM and 100 nM had little effect of SKOV-3 and HEYA8 CSCs [11], and only a partial decrease in sphere formation was observed (Figure 4A). On the other hand, Rb1 or compound K in combination with the same doses of cisplatin or paclitaxel sensitizes these CSCs to both drugs (SKOV-3: Rb1, Rb1 + cisplatin, and Rb1+ paclitaxel, *P* < 0.0001; compound K, compound K + cisplatin, and compound K + paclitaxel, *P <* 0.0001; HEYA8: Rb1, *P* = 0.004, Rb1 + cisplatin, and Rb1 + paclitaxel, *P* < 0.0001; compound K, *P* = 0.004, compound K + cisplatin, *P* < 0.0001, and compound K + paclitaxel, *P =* 0.001) (Figure 4A). In addition, treatment of CSCs isolated from primary ovarian cancer samples inhibited both sphere formation and growth, demonstrating drug-induced death by Rb1 or compound K or in combination with cisplatin or paclitaxel consistently supported the *in vitro* observations (Rb1, CK, Rb1 + cisplatin, Rb1 + paclitaxel, compound K + cisplatin and compound K + paclitaxel, *P* < 0.0001) (Figure 4B).

### Compound K inhibits EMT to exert cytotoxic effects

**Figure 5:**
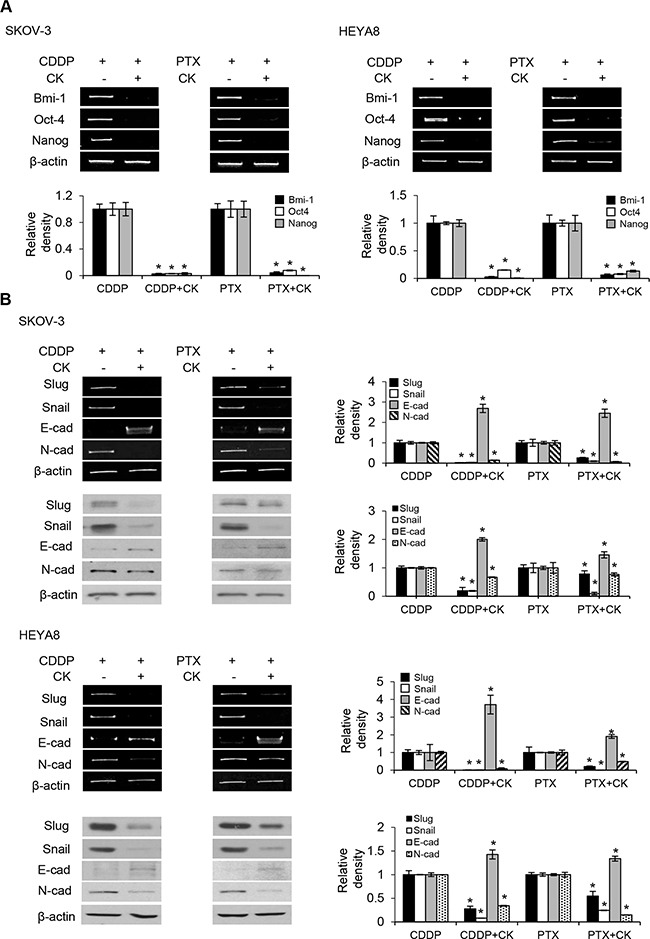
Compound K chemosensitizes CSCs via EMT **A**. Expression of CSC markers Bmi-1, Nanog, and Oct-4 or **B**. EMT markers Slug, Snail, E-cadherin, and N-cadherin were determined by RT-PCR or western blot after using cisplatin (CDDP; 50 μM) or paclitaxel (PTX; 100 nM) alone or in combination with compound K (CK) for 24 h. β-actin serves as an internal control. The signal intensity was determined by densitometry. Data are shown as mean ± SD. *, *P* < 0.05 vs control.

**Figure 6:**
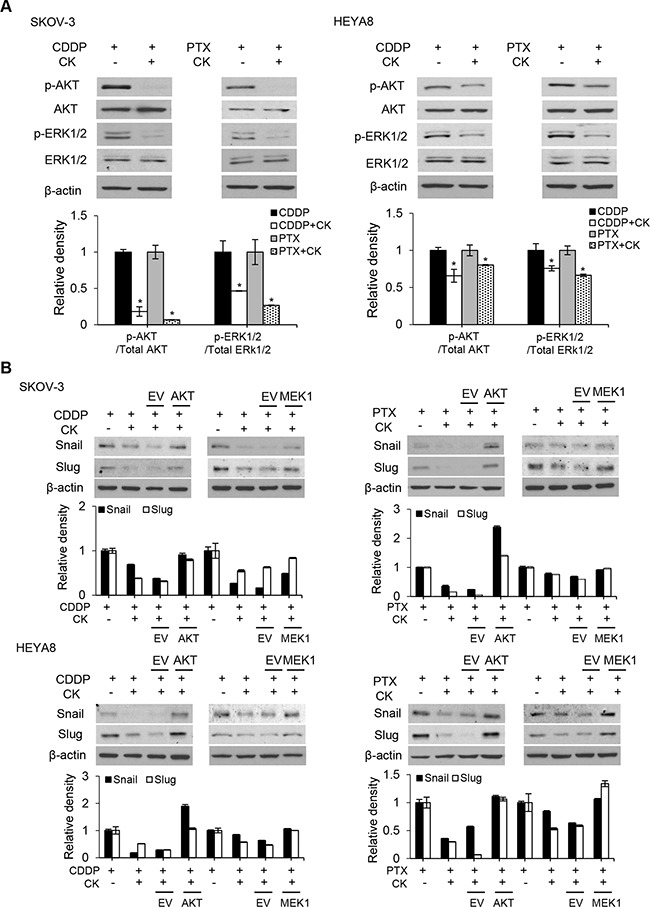
Compound K inhibits EMT through regulating Akt and MAPK signaling pathways **A**. Phosphorylated (active) and total forms of Akt and MAPK family member ERK1/2 were detected by western blot. *, *P* < 0.05 vs control. **B**. Snail and Slug expression were observed after transfection with empty vector (EV), or constitutively active constructs of AKT or MEK1 after treated with CDDP or PTX alone, or in combination with CK. β-actin serves as an internal control. The signal intensity was determined by densitometry. Data are shown as mean ± SD.

Rb1 is readily metabolized to compound K in the gut, and this biotransformation appears to contribute to the reported pharmacological effects of Rb1 [9]. We therefore focus on the molecular mechanisms underlying the action of compound K for the relevance. Emerging evidence suggests an intricate role of EMT in CSC self-renewal [12]. To this end, we first determined the levels of EMT transcription factors. Our results showed that treatment with compound K in the presence of cisplatin and paclitaxel was associated with a marked decrease in the expression of Snail and Slug (Figure 5B). Furthermore, in correlation with our findings from Snail/Slug, we also observed compound K substantially reverted the inhibition of E-cadherin expression, an epithelial marker, and its concomitant increase of N-cadherin expression, a mesenchymal marker (Figure 5B). Similar experiments with Rb1 revealed changes in expression indistinguishable from those in compound K (data not shown). We next determined the molecular mechanisms by which compound K inhibited Snail/Slug expression. Because studies have shown that the PI3K/Akt and ERK1/2 mitogen-activated protein kinase pathways are critical for the activation of Snail/Slug in some cell types [13, 14], we first examined the levels of phosphorylated (active) forms of Akt and ERK1/2 in SKOV-3 and HEYA8 cells treated with compound K. As shown, addition of compound K to the cells significantly decreased the phosphorylation of Akt and ERK1/2 (Figure 6A). To assess the functional significance of compound K-suppressed Akt and ERK1/2 in Snail/Slug expression, we used constitutively active constructs of Akt (E17K) and MEK1 (CA-MEK1). Both Snail and Slug expression were clearly reverted in E17K- and CA-MEK1-transfected cells upon compound K treatment (Figure 6B). These results reveal a key role of Rb1 and compound K in combination with cisplatin and paclitaxel in EMT regulation and show that these compounds also have a role in EMT-regulated CSCs.

### Compound K inhibits the Wnt/β-catenin signaling pathway in CSCs

**Figure 7:**
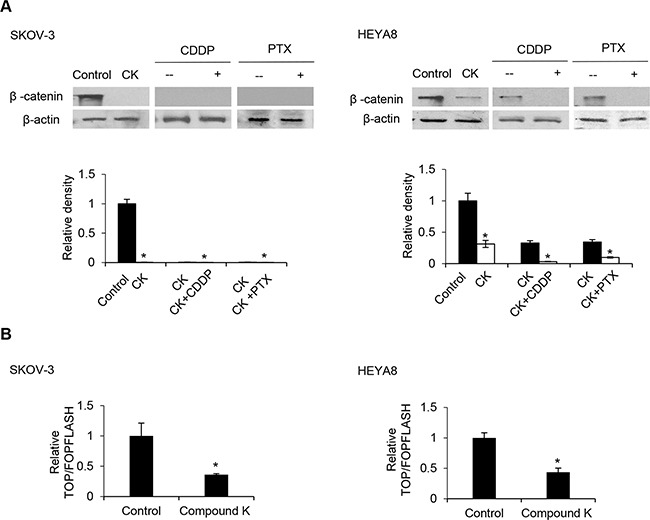
Compound K enhances the chemosensitizing effect through the β-catenin/TCF signaling pathway **A**. Protein levels of β-catenin were analyzed by Western blot and β-actin serves as an internal control. The signal intensity was determined by densitometry. **B**. Cells were transfected with 0.25 μg of either the TOPFLASH or FOPFLASH luciferase reporter constructs containing wildtype and mutant TCF binding sites, respectively, together with 0.5 μg β-galactosidase for normalization of transfection efficiency. Values are normalized luciferase activity (TOPFLASH activity minus the activity devoted to FOPFLASH and normalized to β-galactosidase activity). The absorbance of wells not exposed to compound K (CK) treatment was arbitrarily set as 1. Data are shown as mean ± SD. *, *P* < 0.05 vs control.

**Figure 8:**
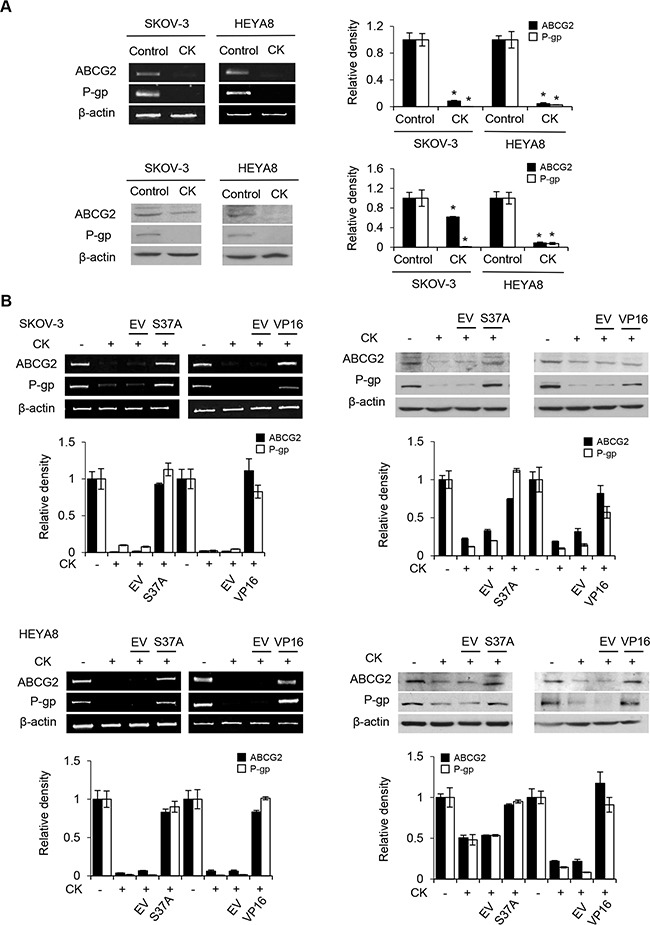
β-catenin activation induces ABCG2 and P-glycoprotein expression **A**. CSCs were treated with compound K (CK) alone. The expression of ABCG2 and P-glycoprotein were determined by RT-PCR and Western blotting, β-actin serves as an internal control. The signal intensity was determined by densitometry. **B**. CSCs treated with CK were transfected with either empty vector (EV), β-catenin S37A, or VP16-TCF construct in the absence or presence of CK. The expression of ABCG2 and P-glycoprotein were determined by RT-PCR and western blot, and β-actin serves as an internal control. The absorbance of wells not exposed to CK treatment was arbitrarily set as 1. Data are shown as mean ± SD. *, *P* < 0.05 vs control.

Next, we inquired the mechanism by which compound K inhibits CSC survival and its possible role in chemoresistance. β-catenin, which plays a pivotal role in self-renewal or tumorigenesis, is a particular intriguing property [15]. Since the elevated levels of β-catenin could direct cell fate [16], we examined the expression of β-catenin in compound K treated CSCs by Western blot analysis. We observed a marked decrease of β-catenin levels upon compound K treatment (Figure 7A). β-catenin is known to interact with T-cell factor (TCF)/LEF to activate the transcription of target genes [17], and evidence exists that correlate increased levels of β-catenin with increased levels of nuclear active β-catenin [18]. Hence, we studied the effect of compound K on the transcriptional activity of β-catenin/TCF using luciferase reporter plasmids encoding multimerized wildtype (TOPFLASH) or mutated (FOPFLASH) TCF binding sites, which have been widely used to characterize β-catenin/TCF-dependent gene expression [19]. In support of the observation, compound K treatment significantly inhibited TOPFLASH activation (Figure 7B) Importantly, compound K led to a sharp decrease in the expression of ABCG2 and P-glycoprotein, two major drug transporters that are implicated in the efflux chemotherapeutic drugs and are targets of β-catenin/TCF [10, 12]. The levels of ABCG2 and P-glycoprotein were reduced 12.8 and 12.5-fold in compound K treatment for SKOV-3, and 22.4 and 38.4-fold in compound K treatment for HEYA8, respectively (Figure 8A). Likewise, the chemosensitizing effect in CSCs by compound K could be reversed by expression of a stable mutant form of β-catenin (S37A) (Figure 8B). There was also a clear decrease of drug-induced cell death by the expression of constitutively active TCF (VP16-TCF) (Figure 8B). These results present the first evidence that compound K inhibit the Wnt/β-catenin signaling in CSC cells, and that these effects were mediated through its downstream CSC-driven effectors ABCG2 and P-glycoprotein.

### Efficacy of compound K and chemotherapy in CSCs *in vivo*

**Figure 9:**
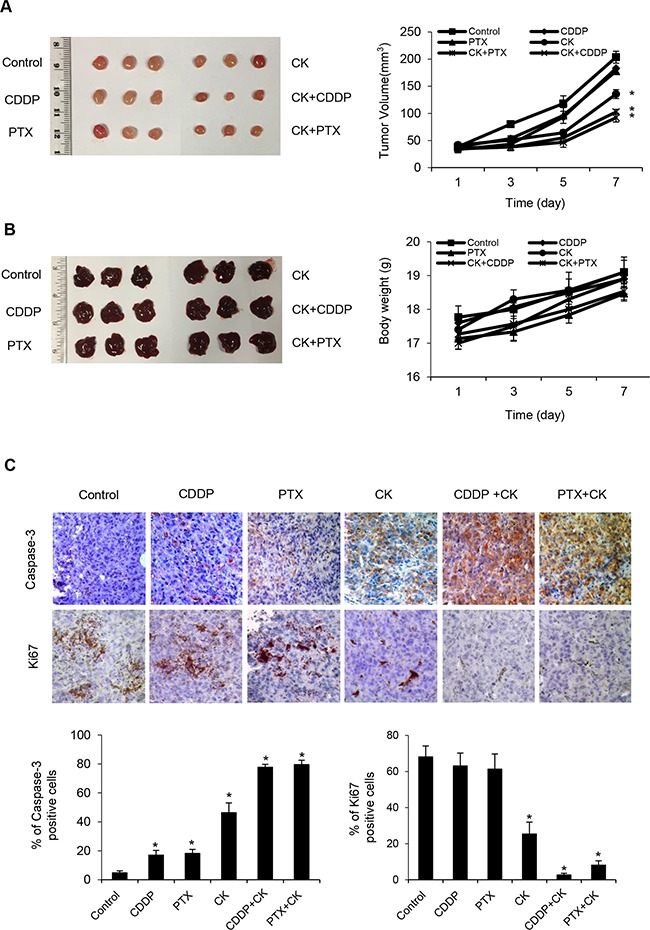
Therapeutic efficacy of compound K Nude mice were injected s.c. with HEYA8 cells (10^6^) and randomly allocated to one of the six treatment groups (n = 3, repeated three times): (i) vehicle, (ii) compound K (CK), (iii) cisplatin (CDDP), (iv) paclitaxel (PTX), (v) CK + CDDP, (vi) CK + PTX. When control animals (vehicle) began to appear moribund (28 days), all animals in an experiment were sacrificed and photographed, and **A**. tumor volume were measured. **B**. Mice weight and macroscopic aspects of characteristic livers were recorded. **C**. Immunohistochemical analysis of caspase-3 and Ki67 expression in tumor tissues (200x). Data are shown as mean + SEM (A, B) and mean ± SD (C). *, *P* < 0.05 vs control.

**Figure 10:**
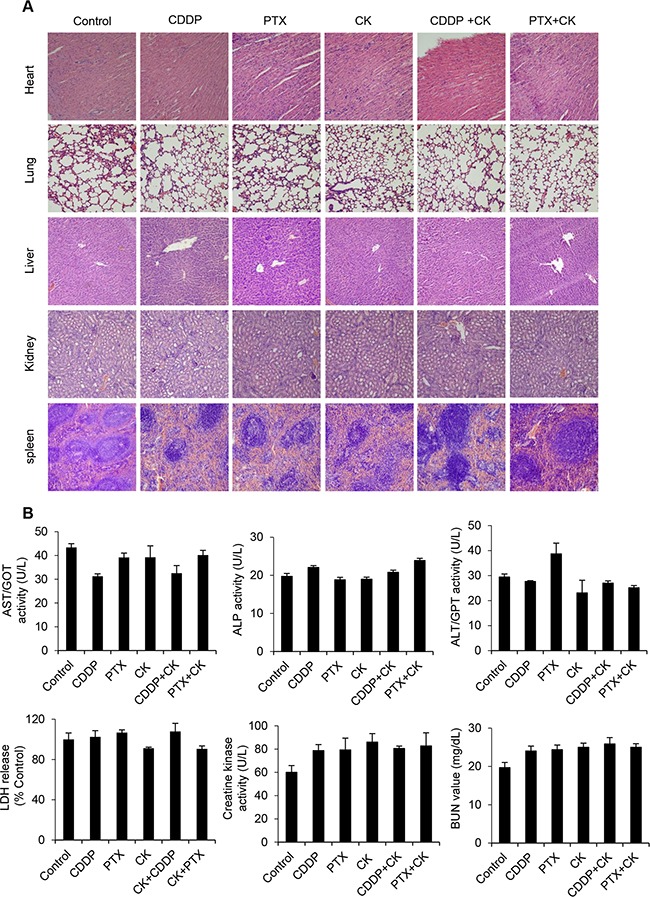
Compound K has no adverse effects **A**. H&E staining of heart, lungs, liver, spleen and kidney tissues (200x). **B**. Peripheral blood was collected from retro-orbital sinus at sacrifice and plasma lactate dehydrogenase (LDH), aspartate aminotransferase (AST), alanine transaminase (ALT), alkaline phosphatase (ALP), creatine kinase and blood urea nitrogen (BUN) were measured and compared to vehicle injected mice. Data are shown as mean ± SD. *, *P* < 0.05 vs control.

To further validate its role *in vivo*, we used mouse xenograft ovarian tumors. Treatment with compound K alone was effective in inhibiting tumor growth compared with vehicle control (Figure 9A). However, treatment with compound K in combination with cisplatin or paclitaxel resulted in even greater reduction in tumor volume (compound K + cisplatin, 50.2%, *P* < 0.05; compound K + paclitaxel, 45.6%, *P* < 0.05). Furthermore, the combination therapy was statistically superior to cisplatin or paclitaxel alone (compound K + cisplatin, compound K + paclitaxel, *P* < 0.05) (Figure 9A). Further, compound K also induced caspase-3 expression *in vivo*, indicating apoptosis activation in compound K-treated HEYA8 xenografts (Figure 9C). These results demonstrate that compound K is potent in suppressing tumor growth from CSCs *in vivo* and that these effects are cytotoxic rather than cytostatic. The compound K regimens were well-tolerated to the tested animals. There was no abnormal behavior or weight loss noted during the entire treatment period (Figure 9B). Moreover, histopathological observations of the liver, heart, kidney, spleen, and lungs in the treated animals showed no histological alterations as compared to the controls (Figure 10A). Blood chemistry showed that compound K did not cause a significant increase in lactate dehydrogenase (LDH), aspartate aminotransferase (AST), alanine transaminase (ALT), alkaline phosphatase (ALP), creatine kinase (CK) and blood urea nitrogen (BUN) (Figure 10B), hence showing that compound K has no adverse effects to the liver, heart, and renal function.

## DISCUSSION

Tumor cell resistance to chemotherapies still forms a major barrier toward effective cancer therapy. This paper presents two important findings. First, Rb1 and its metabolite compound K inhibit CSCs. Second, these compounds enhance cisplatin and paclitaxel anti-cancer effect. These effects are at least in part due to inhibition of the Wnt/β-catenin signaling pathway and EMT. To our knowledge, CSCs chemosensitization has not been documented in ginseng as well as in other traditional Chinese medicine. Our studies represent the first to report the phenomenon and elucidate the signaling mechanisms involved. The combination of these natural products and chemotherapeutic drugs could be of great therapeutic value if included in the treatment regimens.

Ginseng is listed in pharmacopeias and has been widely used in China for medicinal purposes for thousands of years due to its rich content of saponins. Ginsenoside is one of the most widely characterized saponins in the rhizome of ginseng. Ginseng also has a chemopreventive potential in the context of ovarian cancer as seen in a mouse model [5]. For example, it has been demonstrated that ginsenoside Rg3 in combination with cyclophosphamide could improve the living quality and survival time of mice with ovarian tumors [20]. Moreover, case-control studies have pointed toward preventive effects of ginseng products had a preventive effect on ovarian cancer [21]. These results suggest that ginseng might be an ideal cancer chemotherapeutic agent.

Several activities of Rb1 make it desirable both as a therapeutic and as a chemopreventive agent: (a) Unlike other saponins, which often have poor absorption and rapid excretion, Rb1 seems to exhibit a better bioavailability and a desirable duration of action [22]. (b) Rb1 disrupts many of the characteristic cancer promoting activities [23]. (c) In addition, Rb1 has shown to improve the functions of normal cells, such as cardioprotective, hepatoprotective and anti-inflammatory activities, which provide definite advantages than currently used drugs [24–27]. Rb1 has various anti-cancer properties ranging from the inhibition of cell proliferation to angiogenesis as well as in apoptosis induction [28, 29]. Structure-activity analyses suggest that ginsenosides metabolites with less sugar moieties have greater anti-cancer activities. Indeed, among all ginsenosides, compound K (one sugar residue) is found to possess the most potent tumor growth and invasion suppressing activities [30]. We observed that compound K possesses very significant anti-CSC activities as compared to Rb1. The IC_50_ for compound K for inhibiting CSC sphere formation and growth was 125 nM, suggesting that its anti-CSC effect is greater than that of Rb1. The difference in the number of sugar moieties may mediate their different affinities towards molecular targets.

Cisplatin and paclitaxel are two mainstays of anti-cancer drugs in the clinical treatment of ovarian cancer. In addition to the dose-limiting toxicities, their drug resistance poses another major problem for their clinical use [31]. In the present study, we demonstrate that Rb1 and compound K specifically enhances these chemotherapeutic treatments, and that these changes occur at pharmacologically attainable drug levels [11]. Moreover, non-toxic, low doses of cisplatin and paclitaxel with Rb1 and compound K can also induce an enhanced cytotoxic response in the cancer cells (data not shown), suggesting a potential avenue to improve the clinical efficacy. Our findings may also provide a probable explanation as to why Rb1 and its metabolite-containing PPD have substantial benefit in chemoresistant tumors [32]. Imatinib is used in the treatment of a number of cancers, including ovarian cancer [33–36]. We found that Rb1 and compound K had similar efficiency as imatinib in cell growth inhibition and apoptosis induction. Importantly, compound K might imatinib, as it is less sensitive to some resistance mechanisms cancer cells develop [37]. We further identified several mechanisms of action induced by compound K, such as inactivation of ABCG2 and P-gp.

The presence of activated β-catenin in a broad spectrum of human cancers makes Wnt/β-catenin signaling an attractive target for cancer treatment. Wnt/β-catenin signaling has also been implicated in ovarian cancer. While mutations in adenomatous polyposis coli or β-catenin gene are infrequent, an increased β-catenin level and/or altered Wnt/β-catenin signaling are commonly found in different ovarian cancer subtypes and that high β-catenin expression correlates with tumor grade and poor prognosis [38–40]. It is also relevant that Wnt/β-catenin is significantly overexpressed in recurrent lesions [10, 41]. Therefore, the compound K-dependent inhibition of Wnt/β-catenin may hold great value in treating patients with refractory ovarian cancer. ABC transporters are well-established efflux pumps contributing to drug resistance, which are molecular and functional properties of a distinct side population of cells. It has been demonstrated that ABCG2 and P-glycoprotein are the two most important genes for the chemoresistance of CSCs [42–44]. There is emerging evidence that inhibition of Wnt/β-catenin and drug transporters can eradicate CSCs, but to be nontoxic to normal cells/tissue stem cells [45].

There is increasing evidence that the induction of EMT in transformed ovarian cancer cells *in vitro* or in mouse models to generate cells with CSC characteristics, indicating that EMT may be implicated in ovarian cancer aggressiveness [46, 47]. Moreover, CSCs derived from ovarian tumors and metastatic ovarian peritoneal effusions express EMT-associated markers, and that high expression of EMT-inducing genes enhances the metastatic potential for ovarian cancer cells [48]. Our studies reveal a role for Rb1 and compound K in linking EMT and CSCs, which not only shed light on the molecular mechanisms of action but also suggest new therapeutic strategy that warrants clinical investigation.

Therapeutic selectivity is one of the most critical considerations in cancer treatment. Ginseng has been shown to be well-tolerated even at very high doses in patients but nontoxic to normal human cells [49]. We have also shown the differential effects of Rb1 and compound K in ovarian cancer and normal human ovarian surface epithelial cells (Supplementary Figure S1). Pharmacokinetics of Rb1 has not been determined in humans. However, the dosage (250 nM) utilized in this study *in vitro* is similar to the pharmacologically attainable levels of 145-196 nM in the plasma in consuming per gram of Rb1, based on the oral bioavailability of 4.35% of Rb1 in animal studies [50]. Treatment with Rb1 within this dosage range is highly effective in inhibiting ovarian cancer growth and does not seem to exhibit toxicity. Indeed, different phase I trials have shown that Rb1 is safe when consumed at doses as high as 10 g root and a dose of ginsenosides smaller than 300 mg/kg [51, 52].

In summary, this study shows for the first time that ginsenoside Rb1, especially its metabolite compound K, specifically target CSCs via simultaneous inhibition of Wnt/β-catenin signaling and EMT in ovarian cancer. The ability of Rb1 and its metabolite compound K to intervene different cellular pathways in CSCs suggests that Rb1 can be a wide spectrum chemopreventive agent for cancer treatment. These findings are highly clinical significant, and will provide a solid scientific background in the development of Rb1 and compound K in ginseng-based therapeutics for the treatment of relapsed cancers which currently have limited pharmaceutical options.

## MATERIALS AND METHODS

### Cells and cell culture

Human ovarian carcinoma cell lines SKOV-3 and HEYA8 were gifts from Dr. N. Auersperg (University of British Columbia, Vancouver, B. C., Canada) and Dr. J. Liu (MD Anderson Cancer Center, Houston, TX), respectively. Isolation and culture of CSCs from SKOV-3 and HEYA8 or primary human ovarian cancer samples were performed in serum-free stem cell-selective condition as described in Chau *et al*. [10]. For the relapse experiment, control/treated primary spheroids were dissociated and replated as secondary spheroids and imaged daily. Primary tumor samples were obtained from ovarian cancer patients with informed consent and approval by the Taipei Medical University Institutional Review Board. Cisplatin and paclitaxel were purchased from Calbiochem (San Diego, CA) and were used at 50 μM and 100 nM concentration, respectively.

### Reverse transcription and PCR

Total RNA was extracted with Trizol and reversed transcribed, and the cDNA was synthesized using the first-stranded cDNA synthesis kit (Invitrogen). The sequences of the specific primers were as follows: Bmi-1: sense 5’-ATGTGTGTGCTTTGTGGAG-3’, antisense 5’-AGTGGTCTGGTCTTGTGAAC-3’; Nanog: sense 5’-AAGACAAGGTCCCGGTCAAG-3’, antisense 5’-CCTAGTGGTCTGCTGTATTAC-3’; Oct4: sense 5’-ATCCTGGGGGTTCTATT TGG-3’, antisense 5’-TCTCCAGGTTGCCTCTCACT-3’; ABCG2: sense 5’-CTGAGA TCCTGAGCCTTTGG-3’, antisense 5’-TGCCCATCACAACATCATCT-3’; and P-glycoprotein: sense 5’-ACCTGTGAAGAGTAGAACATGAAGA-3’, antisense 5’-AAGATCCATTCCGACCT CGC-3’. β-actin served as a control. The PCR conditions used to amplify Bmi-1, Oct4, ABCG2, and P-glycoprotein were: 33 cycles at 95°C for 30 s, 55°C for 30 s, and 72°C for 30 s. To analyze expression of Nanog, the thermocycling parameters for the PCR reactions were: 35 cycles at 95°C for 30 s, 55°C for 30 s, and 72°C for 30 s.

### Western blot analysis

Equal amounts of cell lysate proteins were separated on 7.5% SDS-polyacrylamide gels and transferred to nitrocellulose membrane. Membranes were blocked for 1 h at room temperature with 5% non-fat milk, and then incubated overnight at 4°C in the primary antibody: β-catenin (1:2000) (Transduction Lab, Lexington, KY), Cleaved caspase-3 (1:1000), caspase-3(1:1000) Snail (1:1000), Slug (1:1000), phospho-Akt (Ser473) (1:1000), total Akt (1:1000), phospho-ERK1/2 (Thr202/Tyr204) (1:1000), total ERK1/2 (1:1000), ABCG2 (1:1000) and P-glycoprotein (1:1000) (Cell Signaling Technology, Beverley, MA). β-actin (1:2000) (Sigma, St. Louis, MO) was used as a loading control. The protein-antibody complexes were detected by horseradish peroxidise-conjugated secondary antibodies (Bio-Rad, Hercules, CA) followed by the enhanced chemiluminescence detection reagents (Amersham, Little Chalfont, UK).

### Proliferation and chemosensitivity analysis

Rates of proliferation and sensitivity to drugs were assessed using the colorimetric MTT (3-(4,5-dimethylthiazol-2-yl)-2,5-diphenyltetrazolium bromide) assay according to the manufacturer's instructions (Sigma, St. Louis, MO). Briefly, 500 μl MTT solution was added to each well and the plate was incubated for 4 h at 37°C. Medium was then aspirated from each well, and 250 μl DMSO was added. Colorimetric analysis was performed at a wavelength of 570 nm using a microplate reader (Bio-Rad, Hercules, CA).

### Trypan blue exclusion assay

Cells were incubated for 5 min at room temperature with an equal volume of 0.4% (w/v) trypan blue solution (Sigma, St. Louis, MO). Cells were counted using a dual-chamber hemocytometer under a light microscope. Viable and nonviable cells were recorded.

### β-catenin/TCF reporter gene assay

Cells were transiently transfected with 0.5 μg of the TOPFLASH or FOPFLASH reporter plasmid (Clontech) using Lipofectamine (Invitrogen). Transfection efficiencies were determined by co-transfection of the β-galactosidase construct. Luciferase and β-galactosidase activity were assayed according to the manufacturer's protocol using the luciferase assay kit from Promega. Luciferase activity in each well was normalized to the β-galactosidase activity. All experiments were assayed in triplicates, and the assay was performed in three independent experiments.

### *In vivo* studies

All animal experiments were approved by the Institutional Animal Care and Use Committee at the University of Hong Kong. 10^6^ cells were subcutaneously (s.c.) injected into the right flank of athymic nude mice. To evaluate the therapeutic effect, treatment was initiated ∼21 days following tumor injection when tumors reached a detectable size 50 mm^3^. Mice (n = 3 per group, repeated three times) were treated by oral gavage, which was relevant to the dietary administration, with either vehicle, compound K (50 mg/kg), cisplatin (5 mg/kg), paclitaxel (2 mg/kg), compound K + cisplatin (50 mg/kg for compound K, 5 mg/kg for cisplatin), compound K + paclitaxel (50 mg/kg for compound K, 2 mg/kg for paclitaxel). Mice were monitored for adverse effects of therapy and sacrificed on day 28 or when any of the mice began to appear moribund. Mice weight and tumor volume were recorded. The length and width of a tumor were measured with a caliper and tumor volume was calculated as [(length+width)/2]^3^. Plasma samples were collected from retro-orbital sinus and further analyzed for hepatic, cardiac, and renal functions including LDH, AST, ALT, ALP, CK, and blood urea nitrogen. Organs including liver, heart, kidney, spleen, and lungs were fixed in formalin for paraffin embedding and stained with hematoxylin and eosin. Ki67 and caspase-3 were also tested by immunohistochemistry.

### Statistical analysis

All data were presented as mean ± S.D. and repeated at least four or five times for *in vitro* studies. Statistical analysis was done using one-way ANOVA followed by Tukey least significant difference *t* test for post hoc analysis (GraphPad Software, San Diego, CA). For *in vivo* studies, all variables were considered nonparametric and presented as mean ± SEM or mean + SD, Kruskal Wallis test was used to evaluate the statistical significance in tumor volume and body weight data differed between control and treatment groups. *P* values of < 0.05 considered statistically significant.

## SUPPLEMENTARY FIGURE


