# Supplementary material for: Ginsenoside-Rb1 targets chemotherapy-resistant ovarian cancer stem cells via simultaneous inhibition of Wnt/β-catenin signaling and epithelial-to-mesenchymal transition
Source: Oncotarget. 2016 Nov 4;8(16):25897–914. doi: 10.18632/oncotarget.13071 (PMC5432225; doi:10.18632/oncotarget.13071)
Supplement: Supplementary file 1 [file oncotarget-08-25897-s001.pdf]

Ginsenoside-Rb1 targets chemotherapy-resistant ovarian cancer stem cells via simultaneous inhibition of Wnt/β-catenin signaling and epithelial-to-mesenchymal transition

SUPPLEMENTARY FIGURE

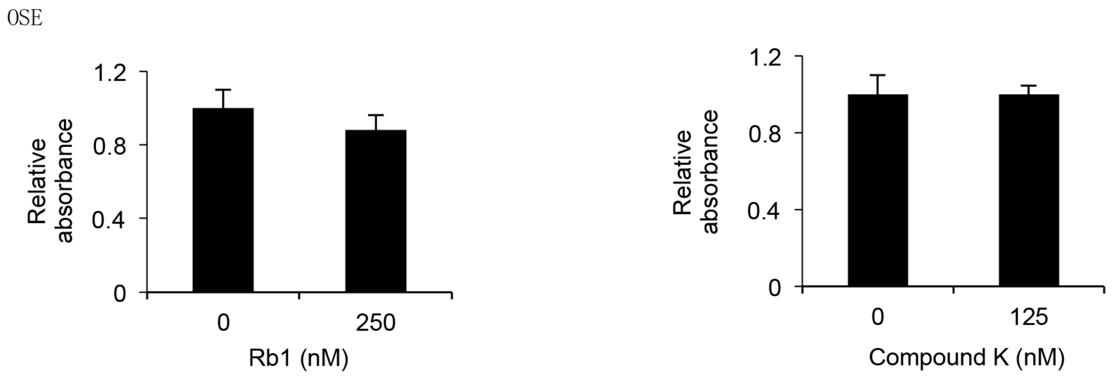

Supplementary Figure S1: No effect of Rb1 and compound K on normal human ovarian surface epithelial cells. Cell viability was determined by MTT assay. The absorbance of wells not exposed to Rb1 or compound K (CK) treatment was arbitrarily set as 1, and cell growth after Rb1 or CK treatment was expressed as the fold changes compared to the control. Experiments were repeated three times, and data are shown as mean ± SD. *, $P < 0.05$ vs control.
